# The relative power of individual distancing efforts and public policies to curb the COVID-19 epidemics

**DOI:** 10.1371/journal.pone.0250764

**Published:** 2021-05-07

**Authors:** Cécile Aubert, Emmanuelle Augeraud-Véron

**Affiliations:** 1 GREThA, University of Bordeaux, Bordeaux, France; 2 TSE, University of Toulouse, Toulouse, France; Frankfurt Institute for Advanced Studies, GERMANY

## Abstract

Lockdown curbs the COVID-19 epidemics but at huge costs. Public debates question its impact compared to reliance on individual responsibility. We study how rationally chosen self-protective behavior impacts the spread of the epidemics and interacts with policies. We first assess the value of lockdown in terms of mortality compared to a counterfactual scenario that incorporates self-protection efforts; and second, assess how individual behavior modify the epidemic dynamics when public regulations change. We couple an SLIAR model, that includes asymptomatic transmission, with utility maximization: Individuals trade off economic and wellbeing costs from physical distancing with a lower infection risk. Physical distancing effort depends on risk aversion, perceptions of the epidemics and average distancing effort in the population. Rational distancing effort is computed as a Nash Equilibrium. Equilibrium effort differs markedly from constant, stochastic or proportional contacts reduction. It adjusts to daily incidence of hospitalization in a way that creates a slightly decreasing plateau in epidemic prevalence. Calibration on French data shows that a business-as-usual benchmark yields an overestimation of the number of deaths by a factor of 10 compared to benchmarks with equilibrium efforts. However, lockdown saves nearly twice as many lives as individual efforts alone. Public policies post-lockdown have a limited impact as they partly crowd out individual efforts. Communication that increases risk salience is more effective.

## Introduction

The COVID-19 pandemic is partly controllable with non-pharmaceutical interventions (NPI) such as physical distancing, especially its most extreme form, lockdown. The latter has curbed the spread of the epidemics during winter 2020 in Wuhan and Shanghai [1, 2], where contacts have been reduced 7- to 8-fold [3]. Vaccination campaigns take time, and new variants introduce additional uncertainty regarding transmission rates, severity and resistance to vaccines. Diverse forms of lockdown remain the main resort to lessen the death toll of the epidemics. When temperatures dropped, large parts of Europe resumed lockdown over the fall and winter. But the associated drastic reduction in activity involves huge economic and welfare costs [4]. The tensions between health and economic impacts lead politics and public opinion to question whether lockdown is necessary or excessive. Opponents to lockdown argue that individuals would take enough precautions if left to choose their behavior. To evaluate this claim, we explicit the interactions between equilibrium effort choices and epidemic spread.

Scientific evidence about the benefits of lockdown does not account for individuals’ efforts. [5] estimate that lockdown has saved 3 million lives in 11 European countries up to May 4, 2020, using constant *R*_0_ values that change with policy interventions. [6] estimate that lockdown-related measures have avoided 500 million cases in in China, South Korea, Italy, Iran, France and the United States between January and April 6, 2020. However, mobility data show that individuals largely adjusted their behavior before restrictions were imposed. Mobility indices negatively correlate with virus reported prevalence (cf. [7, 8], for the US, [9] for France, [10] for Germany). While mobility data is very useful for retrospective analysis, it does not allow the computation of counterfactuals, nor predictions. Using contact matrices [11] provides very precise predictions in stable environments where individuals maintain their usual activities. Our approach complements these: By modeling equilibrium efforts, that are endogenous to the epidemic situation, we can assess the value of an implemented policy (such as lockdown) relative to laissez-faire and predict the impact of others policies. We also provide an analysis of the way behavioral adaptations and epidemic prevalence interact.

To estimate the ‘net impact’ of lockdown, we build a counterfactual scenario in which transmission rates are driven by individual rational choices. We couple an SLIAR compartmental model (with asymptomatic and symptomatic infectious individuals [12]), and a utility maximization model of self-protection under risk. The epidemic model differs from standard ones [13, 14] to reflect the high proportion of infectious individuals with no or only mild symptoms [15]. Asymptomatic transmission is key in determining the effectiveness of public policies [16]. It also makes it difficult to estimate the infection risk. Individuals base their perception on the most salient information available in the media, the number of severe, hospitalized, cases. Distancing decisions are the outcome of a Nash equilibrium: Going out is more or less risky depending on others’ choice to go out. The epidemic transmission rate depends on equilibrium distancing efforts; and thus indirectly on individuals’ risk aversion, costs to avoiding contacts, and beliefs.

Epidemiological parameters are fitted on French data. The start of the first lockdown is very well identified in France (contrary to countries where is was more gradual). French citizens could use information from Italy to better adapt their behavior, since the epidemic pattern was similar, with a 7-day lag. We compute the number of deaths one would have experienced in the absence of lockdown but with individual self-protection efforts, and compare it to simulations based on business as usual and to the actual number of deaths under lockdown. Our results contradict both the large estimates based on business-as-usual and the idea that individual efforts would be as effective as lockdown. We also identify general results about the impact of alternative policies after the end of global lockdown. Public policies that are effective at reducing reported severe cases, induce a reduction in equilibrium efforts. This countervailing effect largely undermines their efficiency. To the contrary, public discourse and measures that convey the gravity of the epidemics situation can potentially lead to quite different epidemics dynamics, since contacts intensity reacts very strongly to perceived salience of the infection risk. Our results indeed show that this perception is a major determinant of the epidemics spread.

## Materials and methods

### Data

We use publicly available data from the French national health agency, covering the period between February 25 and October 30, 2020. The starting date of the lockdown is clearly identified at March 17, 2020: Different policies pertaining to lockdown were taken nearly at the same time (school closure, closure of non-essential business facilities, confinement to a 1-kilometer range from home, outside leisure activities for no more than 1 hour per day…). Data corresponds to different policy stages: pre-lockdown ([*t*_0_, *t*_1_], 02/25—03/17), full lockdown ([*t*_1_, *t*_2_], 03/17—05/11), intermediate lockdown ([*t*_2_, *t*_4_], 05/11—10/30). The intermediate lockdown period is separated into strong restrictions period, which aim to progressively end the lockdown ([*t*_2_, *t*_3_], 5/11–6/17) and a period with fewer restrictions but local constraints ([*t*_3_, *t*_4_], 06/17—10/30, compulsory mask wearing and a curfew being gradually adopted in many areas). On October 30th, corresponding in our modeling to time *t*_4_, a second lockdown is imposed.

**Fig 1:**
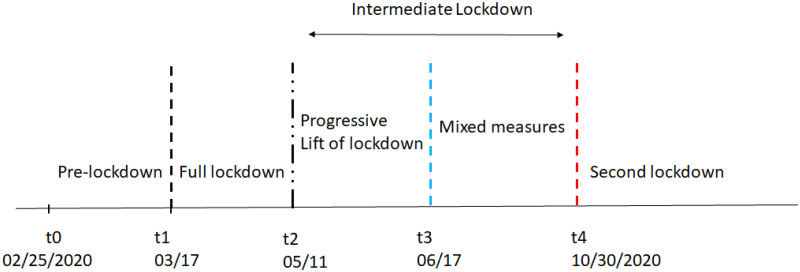
Periods and time intervals.

Fig 1 describes the periods we consider. On our figures, date *t*_1_ is marked by a dashed black line, *t*_2_ by a dash-dot black line, *t*_3_ by a dashed blue line and *t*_4_ by a dashed red line.

Data reporting has been somewhat irregular but the counting method has been consistent until June 3 [17, 18]. These data (in particular daily incidence of hospitalization and daily number of deaths in hospital) are freely available at https://www.data.gouv.fr/fr/datasets/donnees-hospitalieres-relatives-a-lepidemie-de-covid-19/.

### The epidemiological model

We consider an SLIAR compartmental epidemiological model in which the transmission rate of the disease is endogenously defined through an economic model of self-protection choices [19–22]. This epidemic model is widely used to model COVID-19 spread [12, 23–25] because it takes into account a crucial feature of COVID-19: the high proportion of asymptomatic but infectious individuals.

**Fig 2:**
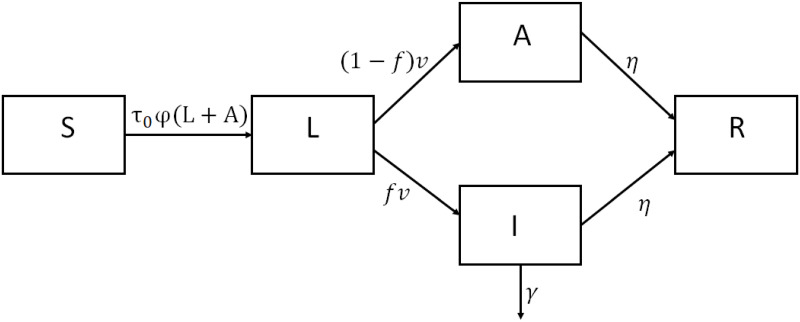
Epidemic diagram.

The epidemic model we consider is depicted in Fig 2.

The population is separated into *susceptible* individuals (in number *S*(*t*) at date *t*) who can get infected, infectious *latent* individuals (*L*(*t*)), *asymptomatic* and mildly symptomatic infectious individuals (*A*(*t*)) and severe symptomatic *infectious* individuals (*I*(*t*)). The latter correspond in our study to hospitalized individuals. In France, up to June 2020, reported cases tested and identified as suffering from COVID-19 were mostly hospitalized individuals, with quite severe forms of the disease. After June, a massive screening campaign began so that the number of reported cases increased largely relative to hospitalized cases. Hospitalized individuals are isolated and no longer play a significant role in disease transmission. Transmission of the disease is due to latent and asymptomatic and mild infectious individuals (in number *L*(*t*)+*A*(*t*)).

At the population level, the dynamics is given by:

$$\begin{array}{ccc} S^{\prime } & = & -\tau_{0}\varphi (t)S(L(t)+A(t))\\ L^{\prime } & = & \tau_{0}\varphi (t)S(L(t)+A(t))-\nu L\\ I^{\prime } & = & f\nu L-(\eta +\gamma (t))I\\ A^{\prime } & = & (1-f)\nu L-\eta A \end{array}$$ (1)

Infected susceptible individuals become latent infectious for an average time *ν*^−1^. Then a fraction 1 − *f* of them become asymptomatic or mild symptomatic infectious; they lose infectiousness after an average time *η*^−1^. The other latent infectious individuals (in proportion *f*) develop a severe form of the disease and become hospitalized; they remain infectious for an average time *η*^−1^ and may also die. Eq 1 is built under the assumption that sojourn time in each compartment is given by an exponential law. In S2 File, we discuss the impact of considering a Gamma distribution, in particular on perceptions. We allow the daily mortality rate to depend on *t*: it highly depends on the processes implemented in hospitals [26], and these processes have evolved during the epidemic spread, with less invasive ventilation techniques. However, given data limitations, we do not account for ICUs capacity constraints. We thus consider that *γ* is a piece-wise constant function, with a first part that prevails until June and a second one, lower than the first, afterwards. The calibration of these values is specified in the results section. We assume that individuals develop full immunity towards SARS-CoV19, and thus do not re-enter the susceptible compartment. This assumption implies that recovered play no role in epidemics transmission. As they also play no role in risk perception, we only need to describe the dynamics for the susceptible and the three infectious compartments. Assuming that individuals develop immunity appears to be appropriate for periods covering a few months (as most of our simulations), given existing studies on immunity [27]. It is however unknown whether immunity persists on a longer time frame. Note that our assumption has consequences on the dynamics as the number of susceptible individuals is always decreasing, and thus in the long run the disease-free equilibrium is the only steady state of the model.

A specificity of our analysis is that the transmission rate *τ*_0_ *φ*(*t*) depends on the infectiousness of the disease but also on individual physical distancing decisions. We denote *τ*_0_ the transmission rate of the disease that would apply in the absence of individual behavior adaptation to the spread of the disease (‘business-as-usual’). This is the transmission rate that can be observed at the beginning of the epidemics. Transmission rate at time *t* is the product of *τ*_0_ times contact intensity *φ*(*t*), defined as the ratio of the number of contacts at time *t* over the number of contacts at the beginning of the epidemic outbreak. This contact intensity models physical distancing.

The aim of this work is to propose a modeling of the contact intensity function under various scenarios and to compare the spread of the epidemics according to these scenarios.

### The behavioral model

In the absence of legal constraints, contact intensity *φ* is set by the self-protection effort *ε* that individuals choose. This self-protection effort *ε* is the outcome of a Nash equilibrium, in which individuals choose a best response to their economic, psychological and epidemiological environment. This methodology is now classical [28, 29] but in most models the authors highlight the impact of the perceived utility and perceived cost of the effort (e.g., vaccination). [30, 31] had highlighted that while epidemic-driven transmission increases in the number of infected individuals, economically-driven transmission decreases with this number, due to higher effort. The new paradigm specific to the COVID-19 epidemics is that, due to latent and asymptomatic transmission, actual prevalence is unknown and perceived prevalence plays a major role. [32, 33] also endogenize transmission to economic incentives, in different and convincing ways, but they consider a classical SIR model with infectious individuals who are aware of their immune status. We incorporate asymptomatic transmission and various preference determinants. We study how effort and epidemic variables interact.

#### Perceived infection risk

Self-protection / distancing effort *ε* induces a reduction in equilibrium contacts intensity. Function $\underline{\varphi }(\varepsilon )\in \left[\varphi_{min},1\right]$ describes the reduction in risk an individual can achieve thanks to her individual choices. We assume that $\underline{\varphi }\left(\varepsilon \right)=1-\varepsilon$. The contact intensity that would prevail in the absence of the epidemics is normalized to 1; The minimal contact intensity achievable is *φ*_*min*_ in ]0,1[.

An essential determinant of the equilibrium self-protection effort is the belief of the individual about her risk of becoming infected. We denote by $p(\varepsilon ,\bar{\varepsilon },\hat{P}\left(t\right))$ the probability describing this belief. It depends on personal exposure (effort *ε*), on the average distancing effort $\bar{\varepsilon }$ in the population, and on perceived prevalence $\hat{P}\left(t\right)$. We assume the following specification, that separates factors controllable via effort ($\underline{\varphi }\left(\varepsilon \right)$) from factors relating to perceived prevalence, which is exogenous from the point of view of the individual:

$$\begin{array}{c} p\left(\varepsilon ,\bar{\varepsilon },\hat{P}\left(t\right)\right)=\underline{\varphi }\left(\varepsilon \right)\pi \left(\bar{\varepsilon },\hat{P}\left(t\right)\right)=\left(1-\varepsilon \right)\pi \left(\bar{\varepsilon },\hat{P}\left(t\right)\right) \end{array}$$

The exogenous element of the belief, $\pi (\bar{\varepsilon },\hat{P}\left(t\right))$, decreases in others’ average distancing effort $\bar{\varepsilon }$ (others’ efforts reduce one’s own risk), and increases in perceived prevalence $\hat{P}\left(t\right)$.

Perceived prevalence $\hat{P}\left(t\right)$ is a function of the salient information available in the media [34], that is: the daily incidence of hospitalization *fνL*. Individuals use information that is not complete (as the number of infectious individuals involved in the disease transmission is not known). The infection risk is however very salient. Perceived prevalence incorporates a weight *k*(*t*), *k*(*t*)>1, that reflects extra attention paid to the COVID-19 risk, awareness that many infectious individuals are undetectable, and over-weighing of small probabilities in self-protection decisions [35]. Perception evolves over time, since there are non measurable variations in content of media, scientific and governmental communication and in general context, that can all affect the salience of the COVID-19 risk over the period we study. Thus perceived prevalence is $\hat{P}(t)=k(t)f\nu L(t)$.

The individual’s belief about her infection risk can therefore be written as $p(\varepsilon ,\bar{\varepsilon },kf\nu L)$. We assume that $\pi \left(\bar{\varepsilon },kf\nu L\right)=\frac{\underline{\varphi }\left(\bar{\varepsilon }\right)k\tau_{0}f\nu L}{\nu +\underline{\varphi }\left(\bar{\varepsilon }\right)k\tau_{0}f\nu L}$.

#### Individual distancing efforts and the corresponding Nash equilibrium

Each individual independently chooses an effort *ε* to reduce her contacts, and thereby reduce her perceived probability of infection at each date. However, reducing contacts creates a disutility, as it involves psychological costs and economic foregone opportunities (like working only very few hours). The chosen self-protection effort *ε* maximizes at time *t* the individual’s expected utility defined as:

$$\begin{array}{c} U\left(\varepsilon ,\bar{\varepsilon },L\right)=\left(1-p\left(\varepsilon ,\bar{\varepsilon },kf\nu L\right)\right)u\left(\varepsilon \right)+p\left(\varepsilon ,\bar{\varepsilon },kf\nu L\right)\lambda u\left(\varepsilon \right) \end{array}$$

where parameter λ measures the loss in utility when becoming sick.

In a large population, each individual considers the dynamics of the epidemic as unresponsive to her own effort: Current effort only affects own immediate risk. Dynamic optimization is thus equivalent to a sequence of instantaneous decisions. We assume that individuals can instantly adjust their distancing behavior, which is very realistic for leisure and shopping activities but less so for work. Introducing a lag in adjustment would be similar to lengthening the latent phase, by creating a longer gap between actual prevalence and behavioral choices.

Function *u*(*ε*) is the von Neumann and Morgenstern utility function. It is concave to represent risk aversion and decreasing in distancing effort *ε*. Because reducing contacts affects economic opportunities and wellbeing when sane, the associated cost is part of the utility function, and is affected by risk aversion. To allow for calibrations, we consider a standard power utility function: $u\left(\varepsilon \right)=\frac{{\left(1-\theta \varepsilon \right)}^{1-1/\sigma }}{1-1/\sigma }$, where *σ* is the constant relative risk aversion (CRRA) parameter. Parameter *θ* measures the utility loss due to contacts reduction: well-being costs (limited activities, confinement in small spaces, isolation, …) and economic costs (reduced work hours and opportunities). Note that in France, at the height of the lockdown only 24% of active individuals were working remotely; 41% [resp. 43%] of workers reported a reduction in their income due to COVID-19 in April [resp. September] 2020 [36].

The impact of reducing own social contacts depends on the average effort undertaken in the population ($\bar{\varepsilon }$). In a large number population, each individual does not take into account how her contacts reduction efforts affects the infection risk of others. Thus, the optimal individual effort *ε* is the best response function

$$\begin{array}{c} BR\left(\bar{\varepsilon };L,k,\sigma ,\theta \right)=\arg\max_{\varepsilon }\left(U\left(\varepsilon ,\bar{\varepsilon },L\right)\right). \end{array}$$

The Nash equilibrium physical distancing effort ε* is determined as a solution of the following equation.

$$\begin{array}{c} \varepsilon^{*}=BR\left(\varepsilon^{*};L,k,\sigma ,\theta \right) \end{array}$$ (2)

In a symmetric equilibrium, because all individuals make their choices in the same way, the average effort within the population is exactly equal to individual effort, but each individual effort is computed taking others’ behavior as given.

We denote the corresponding equilibrium contact intensity $\underline{\varphi }\left({∊}^{*}\left(L\left(t\right)\right)\right)=\varphi^{*}\left(L\left(t\right)\right)$. When needed to highlight the role of parameter *k* (the weight on perceived prevalence), we will write the rational equilibrium contact intensity function as φ*(*L*(*t*);*k*).

## Results

We characterize the equilibrium distancing effort in the absence of lockdown, and use it to contrast the number of deaths under three cases: full lockdown (data), ‘business-as-usual’, and equilibrium effort (counterfactual). We then assess the impact of the prolonged intermediate lockdown phase on epidemic dynamics given individual choices and latent and asymptomatic transmission.

### Equilibrium distancing effort

The individual physical distancing effort is a best response to others’ average behavior $\bar{∊}$, and is given by $BR(\bar{\varepsilon })$:

$$\begin{array}{c} BR\left(\bar{\varepsilon }\right)=\min\{\max\{\frac{1}{2\sigma -1}(\frac{\sigma }{\theta }-\left(\sigma -1\right)\frac{1+\lambda k\tau_{0}\left(1-\bar{\varepsilon }\right)fL}{\left(1-\lambda \right)k\tau_{0}\left(1-\bar{\varepsilon }\right)fL}),0\},1-\varphi_{min}\} \end{array}$$

The symmetric Nash equilibrium is the solution to equation ε* = *BR*(ε*) (i.e., all individuals best-respond to average efforts), under the constraints that this solution lies in [0, 1 − *φ*_*min*_]. It can be explicitly computed as

$$\begin{array}{c} \varepsilon^{*}=\max\{\min(E,1-\varphi_{min}),0\} \end{array}$$ (3)

where

$$\begin{array}{ccc} E & = & -\frac{(2\sigma -1)\lambda -\sigma }{1-\lambda }+\frac{\sigma }{\theta }\\ & & -\sqrt{{\left(-\frac{(2\sigma -1)\lambda -\sigma }{1-\lambda }+\frac{\sigma }{\theta }\right)}^{2}-4(\left(2\sigma -1\right)(\frac{\sigma }{\theta }+\frac{1-\sigma }{1-\lambda })+\frac{\left(2\sigma -1)(1-\sigma \right)}{k\tau_{0}fL})} \end{array}$$

All parameters change the equilibrium physical distancing effort in the expected direction. Their impact is complex and quite different from proportionality. Equilibrium effort increases non linearly in *risk aversion* *σ*, and in *perceptions* about disease severity λ and about perception weight *k*. It decreases non linearly in *personal economic and welfare costs* *θ*. These costs represent lost economic revenues and opportunities as well as psychological costs from isolation. It is noticeable that their impact on effort, while monotonic, takes a complex form, so that multiplicative approximations of their effect would be inadequate.

#### Distancing effort by age group

We analyze in S3 File the case with three age classes (children, young adults and older adults), where the two adult classes choose their effort. Mortality risks and general mobility differ significantly only after age 65 (our ‘older adults’ class). The distancing effort of children is determined by exogenous factors, e.g., sanitary regulations and school opening decisions. COnsidering different age classes increases the number of parameters and, more importantly, the number of equilibrium configurations (the equilibrium effort in each age class and the average in the population are bounded by 1 − *φ*_*min*_ and by 1. An equilibrium can involve any mix of internal and bounded solutions). We cannot use age classes in the remainder of the analysis as the economic data to calibrate the model is not available (S3 File). The theoretical model however provides some insights: When the disease is (perceived as) severe, equilibrium efforts from the young and the old are *substitutes*, and *decrease* in children’ average effort and proportion. School closure, by increasing this average, would lead to lower efforts from the other age groups. This would erode some of the benefits of school closure in terms of transmission between children, and forced distancing for homeschooling parents.

### The impact of lockdown on number of deaths

We study the epidemic dynamics under various scenarios on contact intensity during lockdown [*t*_1_, *t*_2_]. The list of epidemiological parameters is given in Table.1 in S1 File. The counterfactual situations we consider enable to better understand the net impact of lockdown on the number of deaths. We compare the counterfactual dynamics given by our rational equilibrium physical distancing model to simulations based on business-as-usual (no reduction in contacts intensity in comparison to what they were before the epidemic outbreak, i.e., *φ* = 1) and to the actual lockdown situation, fitted by a time dependent function on the data.

Our methodology is the following. We first use the data to calibrate an exogenous time dependent contact intensity function for the lockdown period. From our behavioral model, we can then compute the corresponding disease perception weight *k*. Then we use these calibrated values of *k* to run the counterfactual scenarios.

#### Calibration of lockdown on the data

The impact of lockdown on contact intensity is modeled with a time dependent function *φ*_*Loc*_, defined by System 4. Function *φ*_*Loc*_ is set to 1 for the period before public health measures had been taken: [*t*_0_, *t*_1_]) (with *t*_1_ = March 17). The impact of these interventions is not instantaneous [37]. An exponential form can model this delay, as in [38] for the 2014 Ebola virus outbreak or [24] for COVID-19. But because the French lockdown was very long (9 weeks in its strictest form), we use a form that has a better fit, similar to [37], where *φ*_*Loc*_(*t*) decreases from 1 to *a* > 0. Function *φ*_*Loc*_(*t*) is given by Eq 4.

$$\begin{array}{c} \varphi_{Loc}(t)=\{\begin{array}{c} 1\;\text{for}\;t<t_{1}\\ a+\left(1-a\right)e^{-\mu (t-t_{1})},\;\text{for}\;t>t_{1} \end{array} \end{array}$$ (4)

Parameters *a*, *μ* have been calibrated with least-squares fitting. We also use the lockdown simulation and the data to fit mortality rate *γ*. The calibration resulted in: *a* = 0.1698953, *μ* = 0.1713832 and *γ* = 0.0278991 day^−1^. The value we obtain for the mortality rate is consistent with other studies [39]. Moreover, contacts have been reduced 5-6 fold in France during lockdown, as compared to 7-8 fold in Wuhan and Shanghai [40]; this is also consistent since the French lockdown was comparatively less strict than in China. Because the first lockdown in France was still very restrictive for the population, we assume that the lowest value of contact intensity achieved during this lockdown is the incompressible level, i.e., the minimum value that can be taken by the contact intensity function: we set *φ*_*min*_ = *a*.

#### Counterfactual scenario with equilibrium efforts

Our characterization of equilibrium effort is used to simulate the counterfactual epidemic dynamics if there had been no lockdown, but rational individual self-protection. The epidemiological dynamics are given by System 1, with counterfactual contact intensity function *φ*(*t*) = *φ*_*CF*_(*t*) defined as

$$\begin{array}{c} \varphi_{CF}(t)=\{\begin{array}{c} 1\;\text{for}\;t<t_{1}\\ \varphi^{*}\left(L\left(t\right);k\left(t\right)\right)\;\text{for}\;t>t_{1} \end{array} \end{array}$$ (5)

Parameters in the utility function are set at *θ* = 0.1, λ = 0.1, and the CRRA coefficient of risk aversion, *σ*, at *σ* = 1.5 [41].

We use Eq 2 together with contact intensity *φ*_*Loc*_ and the value of *L*(*t*) (computed using system 1 and function *φ* = *φ*_*Loc*_) to characterize the values of parameter *k*(*t*) during the lockdown.

$$\begin{array}{c} k=\frac{1-\sigma }{f\tau_{0}L\left(t\right)\left(\left(2\sigma -1\right)\left(1-\varphi_{Loc}\left(t\right)\right)-\frac{\sigma }{\theta }\varphi_{Loc}\left(t\right)\right)\left(1-\lambda \right)+\left(\sigma -1\right)\lambda \varphi_{Loc})} \end{array}$$ (6)

**Fig 3:**
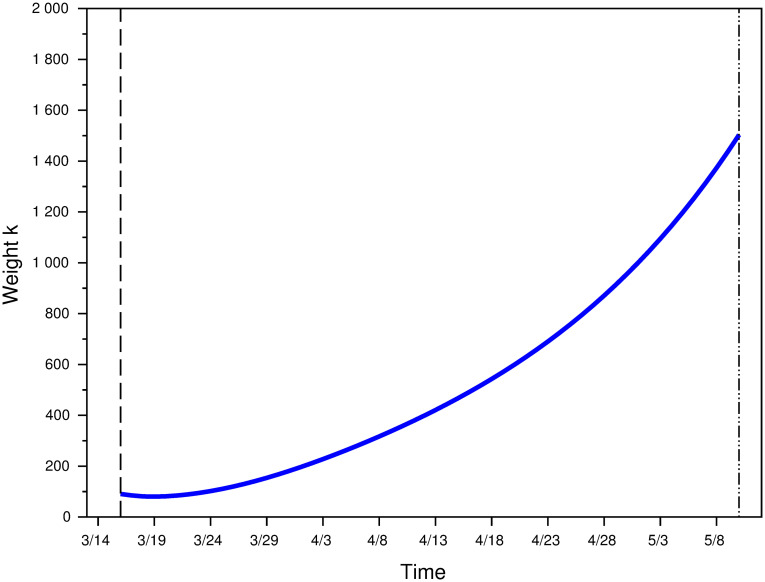
Computed value of weight k during the lockdown period [*t*_1_, *t*_2_].

Fig 3 represents this effective value of weight *k* over the lockdown period [*t*_1_, *t*_2_], using the data to compute its actual values. It varies from *k* = 90.66 at the beginning of lockdown to *k* = 1503.36 at the end of the lockdown period. S2 File similarly computes the value of *k* that would best fit the data with a 2-parameter Erlang distribution, and shows that it would be higher than the one here, but that the relative difference decreases in prevalence.

#### Contact intensity under lockdown vs. under equilibrium effort

**Fig 4:**
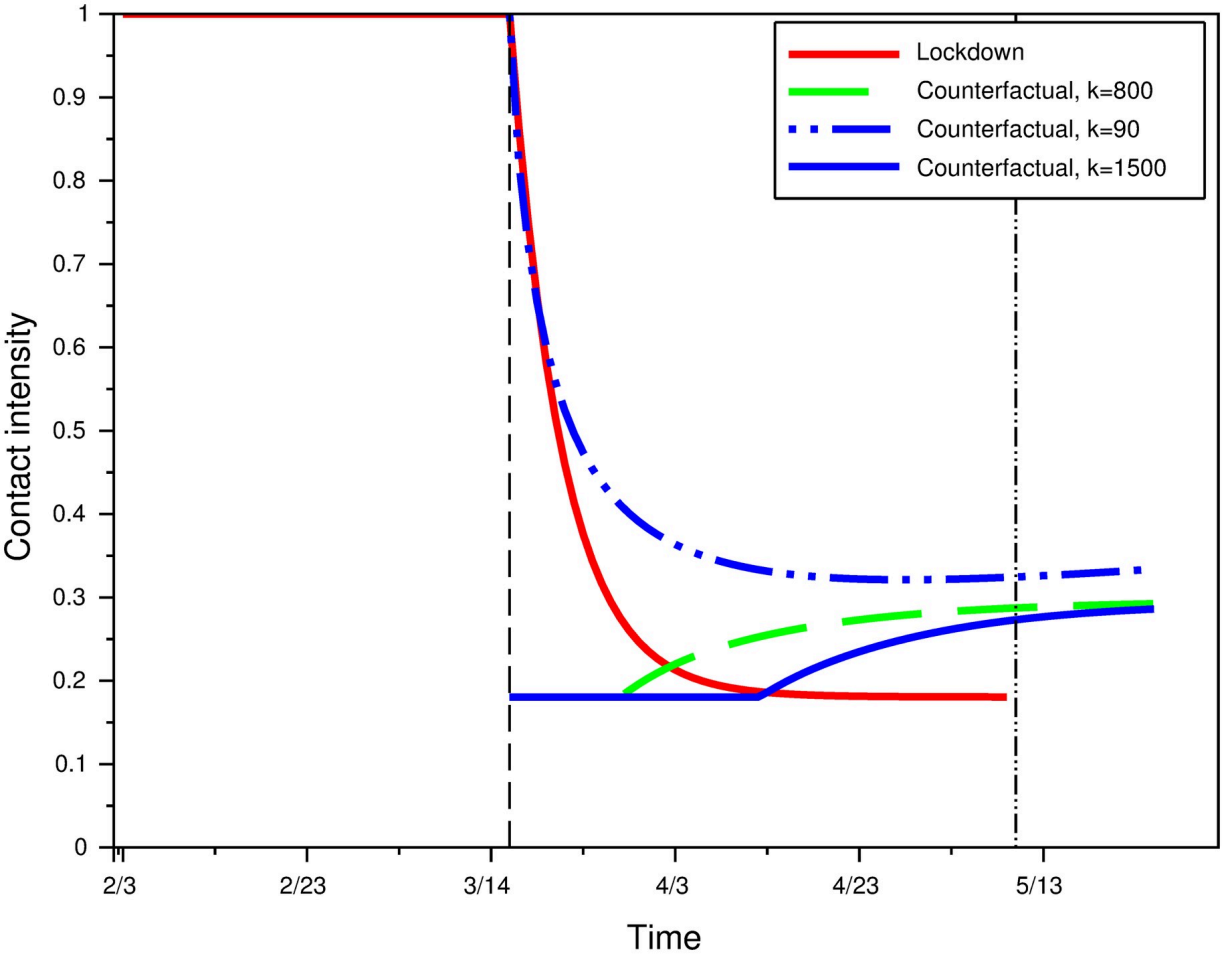
Contact intensity under actual lockdown, and counterfactual rational equilibrium contact intensity for different prevalence weights *k*.

Fig 4 represents contact intensity under various scenarios. Function *φ*_*Loc*_(*t*) is plotted to represent contact intensity during the lockdown period (red line). Function φ*(*L*;*k*) is plotted to model contact intensity under rational equilibrium, where *L* is endogenously determined using System 1 together with function *φ*(*t*) = *φ*_*CF*_(*t*) for various values of *k*. Business-as-usual corresponds to a contact intensity equal to 1 by definition.

For a weight *k* remaining constant at the same level as at the start of the lockdown period (*k* = 90), contact intensity in the counterfactual model is always larger than the actual contact intensity during lockdown. For a large enough weight however (*k* = 800 or 1500), individuals immediately reduce their contact intensity to is minimal value. As a consequence, the number of infected individuals becomes very low in the dynamical system 1, and contact intensity increases after a while as a consequence. This strong feedback mechanism between infectives and distancing effort is consistent with survey data from Datacovid / Ipsos: the latter shows a weakening of distancing efforts after the end of lockdown, when prevalence was very low. At the end of the lockdown, the average (self-reported) number of close contacts increased from 4.5 the first week to 5.8 the second week up to 7.1 between May 26 and May 31 [36] (data available at www.datacovid.org). This increase in the number of contacts remains however low compared to the contact intensity prevalent at the beginning of the epidemics.

#### Avoided deaths under lockdown and equilibrium effort

To provide an assessment of the number of lives saved thanks to lockdown, we compute the number of deaths in hospital under various scenarios using dynamical system 1. COVID-19 results in death in hospital but also in retirement homes and at home, which we do not take into account in our model. The only data we can use for simulations is indeed that on deaths at hospitals: Deaths in retirement homes have been announced in bulk, and death certificates for deaths at home can take months to be included in the data [18]. We therefore need to extrapolate data from the lockdown period in order to compute the total number of deaths. Using data on the number of deaths at home (released on August 23, 2020) [17] and in retirement homes during lockdown, we find that the total number of deaths is 1.45 times the number of deaths at hospital.

**Fig 5:**
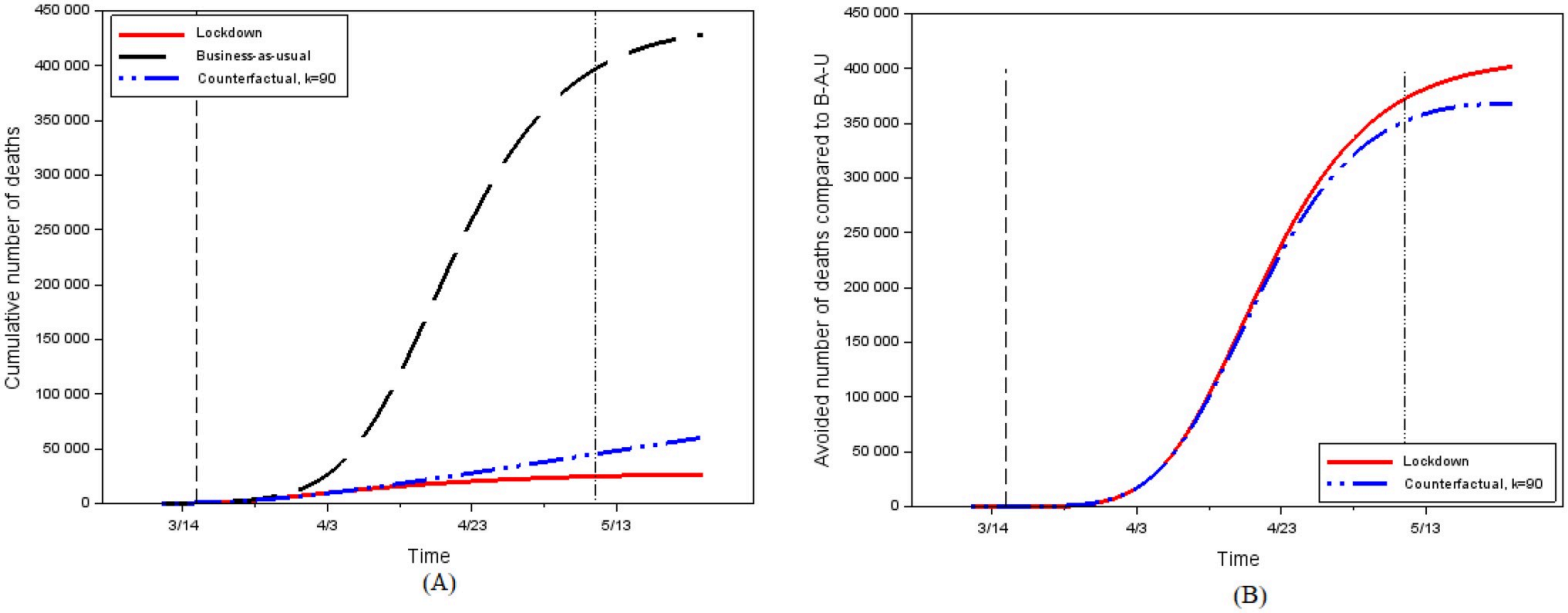
The impact of lockdown. (A) Cumulative number of deaths under various scenarios. (B) Avoided deaths compared to business-as-usual.

Fig 5 contrasts the cumulative number of deaths under various scenarios. Fig 5A shows the number of deaths in the actual situation (lockdown beginning on March 17), in the business-as-usual situation (if people had behaved as before the epidemics outbreak), and in the rational self-protection equilibrium model for the weight parameter *k* taken to be as it prevailed at the beginning of the lockdown period (*k* = 90). To better represent the number of deaths avoided with lockdown and with equilibrium effort, Fig 5B graphs the difference between the cumulative number of deaths in the business-as-usual case and the other situations.

Lockdown appears to have saved a total of nearly 400,000 lives compared to business as usual. And because we cannot account for ICUs saturation, we may underestimate the total number of deaths in the “business-as-usual” scenario, where the peak number of cases is very high. However, this scenario seems unrealistic as it does not accounts for the behavioral changes that would have been observed in the absence of lockdown. Our counterfactual based on endogenously determined effort indeed provides very different results.

Lockdown corresponds to fewer than 25000 deaths over its duration, while unconstrained equilibrium efforts (with *k* = 90) would have lead to 45,200 deaths. Using business-as-usual as a benchmark leads therefore to a very large overestimation of casualties. Nevertheless, compared to lockdown as actually implemented, equilibrium efforts with *k* = 90 would have lead to nearly twice as many deaths. Moreover, the public announcement of the lockdown, done 5 days before starting date *t*_1_, may have had an impact on parameter *k*, by credibly conveying the gravity of the epidemic situation. The value for *k* could therefore have been even smaller than 90 in the absence of a lockdown (our computations show that for *k* = 15 and *k* = 30, the counterfactual model would have led to resp. 7.5 and 4.6 times more deaths than lockdown). Lockdown has not saved as many lives as sometimes claimed but it has had a very large impact nevertheless and is in no way comparable to an appeal to freely chosen individual efforts.

### Gradually lifting restrictions to prevent immediate rebounds

We now study the period between the end of full lockdown (*t*_2_, May 11) and the introduction of a second lockdown (*t*_4_, October 30), in order to assess whether an intermediate period with remaining constraints has helped contain the epidemics. After a long lockdown period, prevalence is very low and lifting restrictions is especially attractive. Our SLIAR behavioral model however shows that a sudden lift of all restrictions could have led to a rebound, exactly—and paradoxically—because prevalence is low, so that equilibrium effort plummets. French data provides interesting evidence, because many restrictions remained imposed after full lockdown ended (May 11). An *‘intermediate lockdown’* has been instituted with strongly recommended remote work, compulsory mask wearing and priority rules for essential workers in public transportation, very limited school reopening and a 100-km limit on travel. These institutional intermediate constraints were officially relaxed on June 17, which we denote *t*_3_ and plot in the figures with a vertical blue dashed line.

Our methodology is the same as in the previous section. We first determine an exogenous time-dependent contact intensity function, *φ*_*I*_(*t*), that fits the data. We then use this calibration to compute the corresponding weight *k* (Eq 6). This parameter, that comes from the data, is then used to run the rational equilibrium model and compute the number of deaths in two scenarios for period *t*_2_—*t*_4_: i) An ‘actual scenario’ which corresponds to the actual epidemic spread, calibrated on data and described by the function *φ*(*t*) = *φ*_*ILoc*_(*t*), and ii) a ‘counterfactual scenario’, described by a function *φ*(*t*) = *φ*_*CFILoc*_(*t*). In the ‘actual scenario’, social distancing remains constrained and given by the same function (*φ*_*Loc*_, fitted on the data) as during lockdown up to *t*_3_; the behavioral model is then applied from *t*_3_ to *t*_4_. In the ‘counterfactual scenario’, the behavioral model is set immediately after the end of the full lockdown, from time *t*_2_, to reflect that individuals would have been free to choose their distancing effort immediately after the end of full lockdown.

#### Calibration of the intermediate lockdown period on the data

Function *φ*_*I*_(*t*) is defined as follows and models contact intensity from time *t*_2_ to time *t*_4_.

$$\begin{array}{c} \varphi_{I}(t)=\{\begin{array}{c} \varphi_{Loc}\left(t\right)\;\text{for}\;t<t_{3}\\ \frac{a(1+be^{ct_{3}})}{\left(1+be^{ct}\right)}\;\text{for}\;t>t_{3} \end{array} \end{array}$$

**Fig 6:**
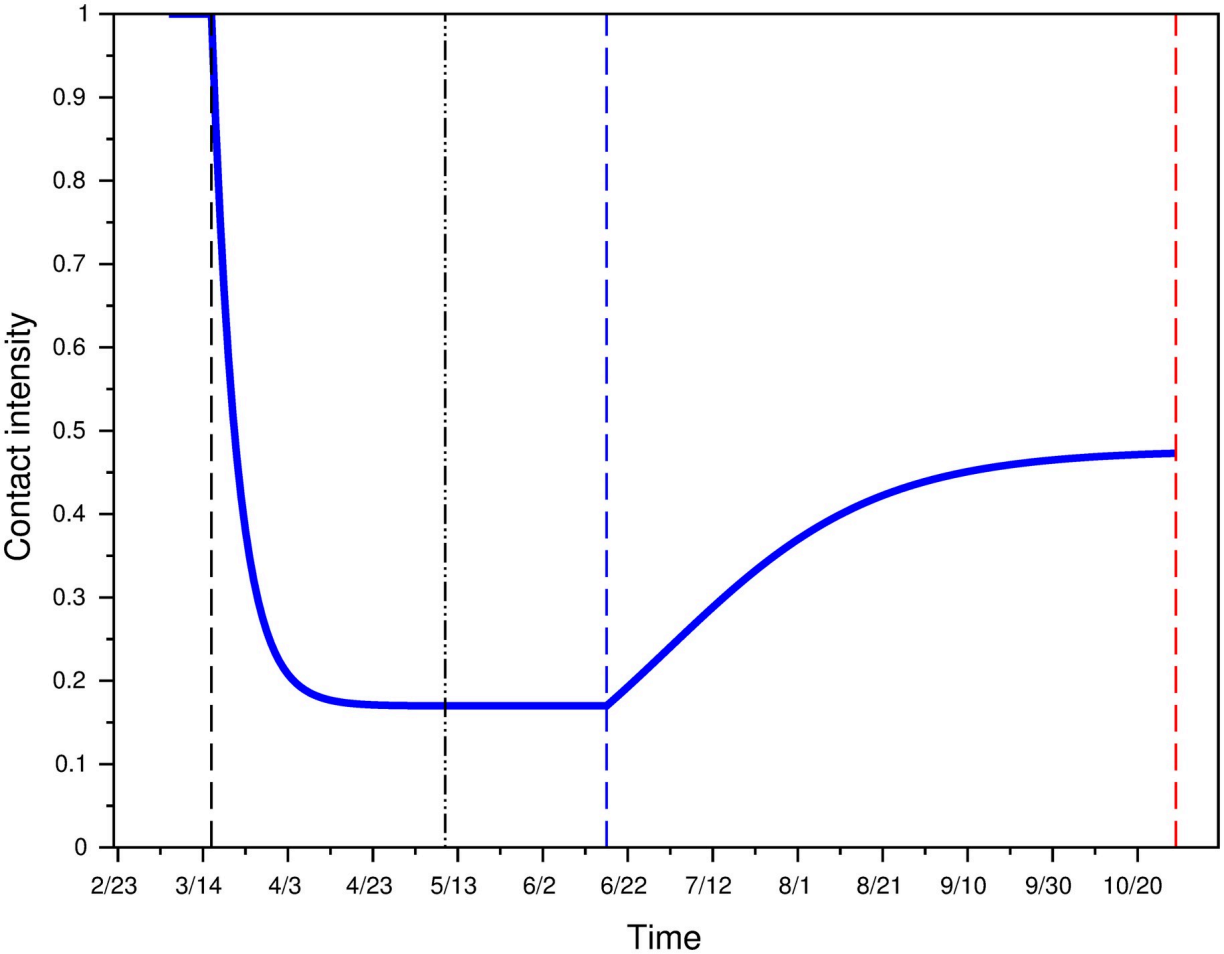
Contact intensity modeling up to Oct. 30.

Fitting the data with least square methods yields *a* = 0.1698953, *b* = 454.0929 and *c* = −0.0406419. Fig 6 shows the actual contact intensity during the intermediate lockdown period. It can be seen that contact intensity has strongly increased in July and mid-September before stabilizing. By October 30, contact intensity is 0.47.

**Fig 7:**
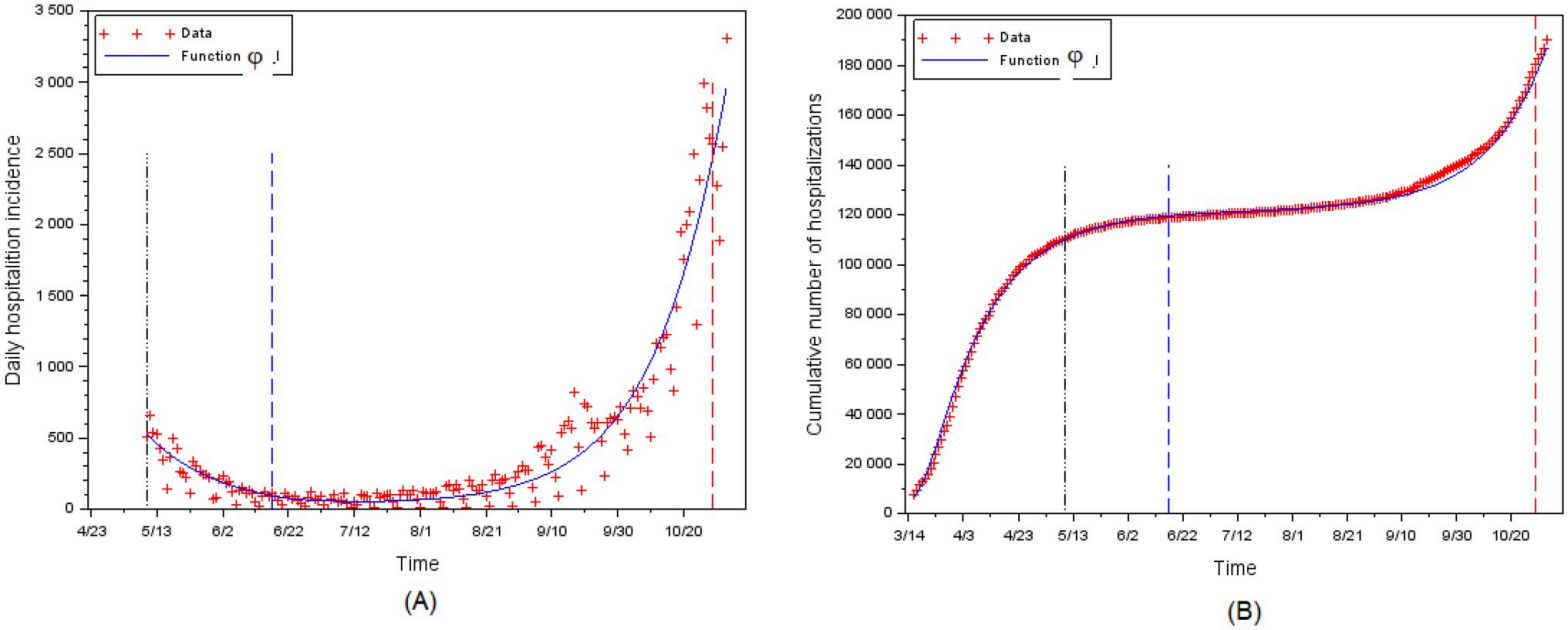
Calibration of daily hospitalizations. (A) Incidence. (B) Cumulative.

The mortality rate in hospital has declined: least square methods yield *γ* = 0.0165696 for *t* > *t*_2_ (cf. Fig 7). This is consistent with [26] according to which the medical practices that are now used in ICU enabled to reduce the mortality rate in hospital.

#### Modeling with rational self-protection equilibrium

The contact intensity function for the ‘actual scenario’ used for [*t*_2_, *t*_4_] is *φ*(*t*) = *φ*_*ILoc*_(*t*), defined as follows.

$$\begin{array}{c} \varphi_{ILoc}(t)=\{\begin{array}{c} \varphi_{Loc}\left(t\right)\;\text{for}\;t<t_{3}\\ \varphi^{*}\left(L;k\left(t\right)\right)\;\text{for}\;t>t_{3} \end{array} \end{array}$$ (7)

**Fig 8:**
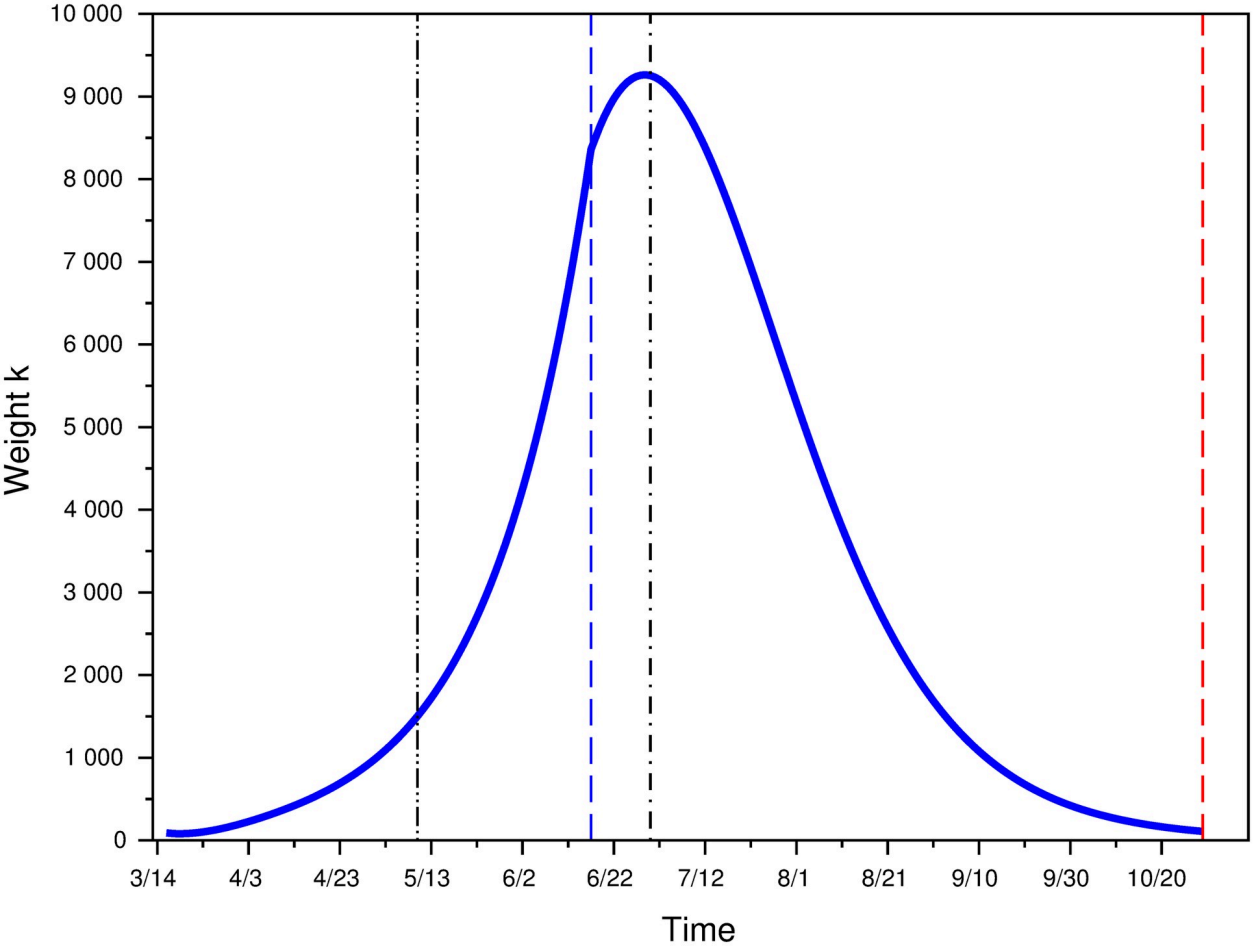
Weight *k* up to Oct. 30.

Using this expression, function *φ*_*I*_(*t*) and Formula 6, we can compute the actual weight parameter *k* that corresponds to the data. Fig 8 represents the evolution of *k* during the intermediate lockdown period.

As during the lockdown period, weight perception *k* is time dependent. It increases up to the July 1, and then decreases up to October 30, where the value of *k* reaches 108.94, which is quite similar to its value at the beginning of the first lockdown.

#### Counterfactual: Assessing the impact of intermediate lockdown using the behavioral model

We have assumed in the ‘actual scenario’ that the measures that have been taken after lockdown prevented the behavioral model to fully apply between time *t*_2_ and time *t*_3_. In the ‘counterfactual model’ we now consider, we assume that individuals are free to choose their level of contacts so that the behavioral model applies from time *t*_2_ onward. We model contact intensity as given by Eq 8 as follows.

$$\begin{array}{c} \varphi_{CFILoc}(t)=\{\begin{array}{c} \varphi_{Loc}\left(t\right),\;\text{for}\;t<t_{2}\\ \varphi^{*}\left(L;1500\right),\;\text{for}\;t>t_{2} \end{array} \end{array}$$ (8)

**Fig 9:**
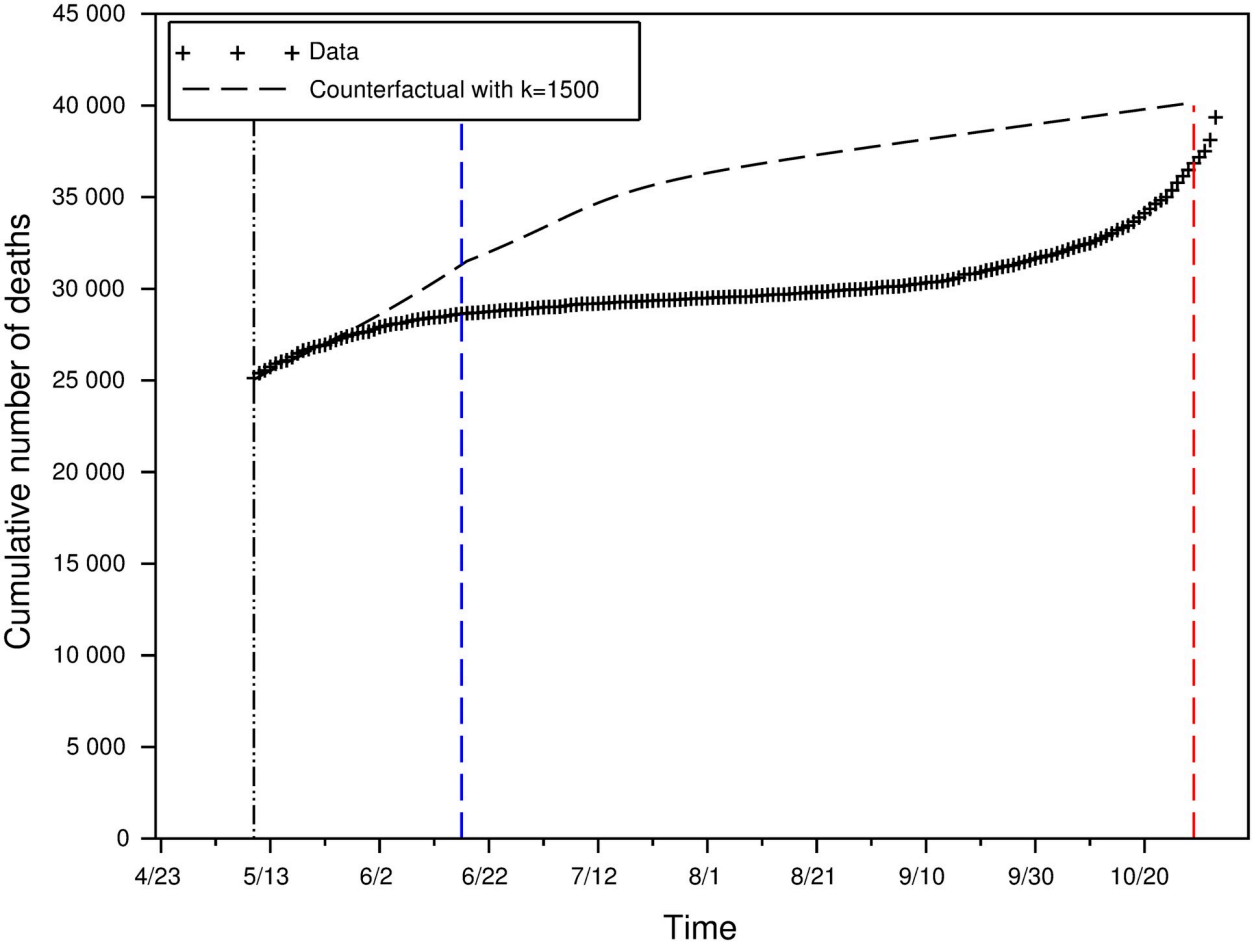
Cumulative number of deaths during intermediate lockdown (data) vs in the equilibrium counterfactual.

Fig 9 plots the counterfactual dynamics obtained through function *φ*_*CFILoc*_, that is: assuming that individuals had been free to behave as in the rational equilibrium immediately at date *t*_2_.

By July 1, the total number of deaths was 29,861. In the counterfactual model, the total number of deaths at the same date would have been 33,058. Thus the very progressive lifting of lockdown saved around 3,200 lives.

## Discussion

Equilibrium effort continuously adjusts to the number of daily hospitalizations, creating complex epidemic dynamics. We use our model of behavior to analyze the interplay between rational equilibrium contact intensity and various public policies after the beginning of the second lockdown (*t*_4_ = October 30, 2020). The conditions for this second lockdown are drastically different from the ones of the first lockdown, with weaker constraints (e.g., schools are still open and remote work is not mandatory in many sectors). Our simulations are not meant to be exact quantitative predictions, given large remaining uncertainties on the consequences of this lockdown. We highlight general patterns and complex interactions, between individual maximization, epidemic prevalence and policy effectiveness.

To fit the data, we assume that for *t* ∈ ]*t*_2_, *t*_3_[(intermediate period), contact intensity is modeled using *φ*(*t*) = *φ*_*I*_(*t*). The subsequent transmission rate *φ*(*t*) for *t* > *t*_3_ is determined by our behavioral assumptions, with adjustments to represent specific policies.

### Distancing effort, perceptions and public salience

A main result of our SLIAR behavioral model is that, in the absence of a full lockdown, individuals adjust their rational equilibrium contact intensity according to epidemic variables. However, this choice of contact intensity also has consequences on the spread of the epidemics. We first study how this spread depends on individual perceptions about the disease.

#### Contact intensity and perception weight on prevalence

**Fig 10:**
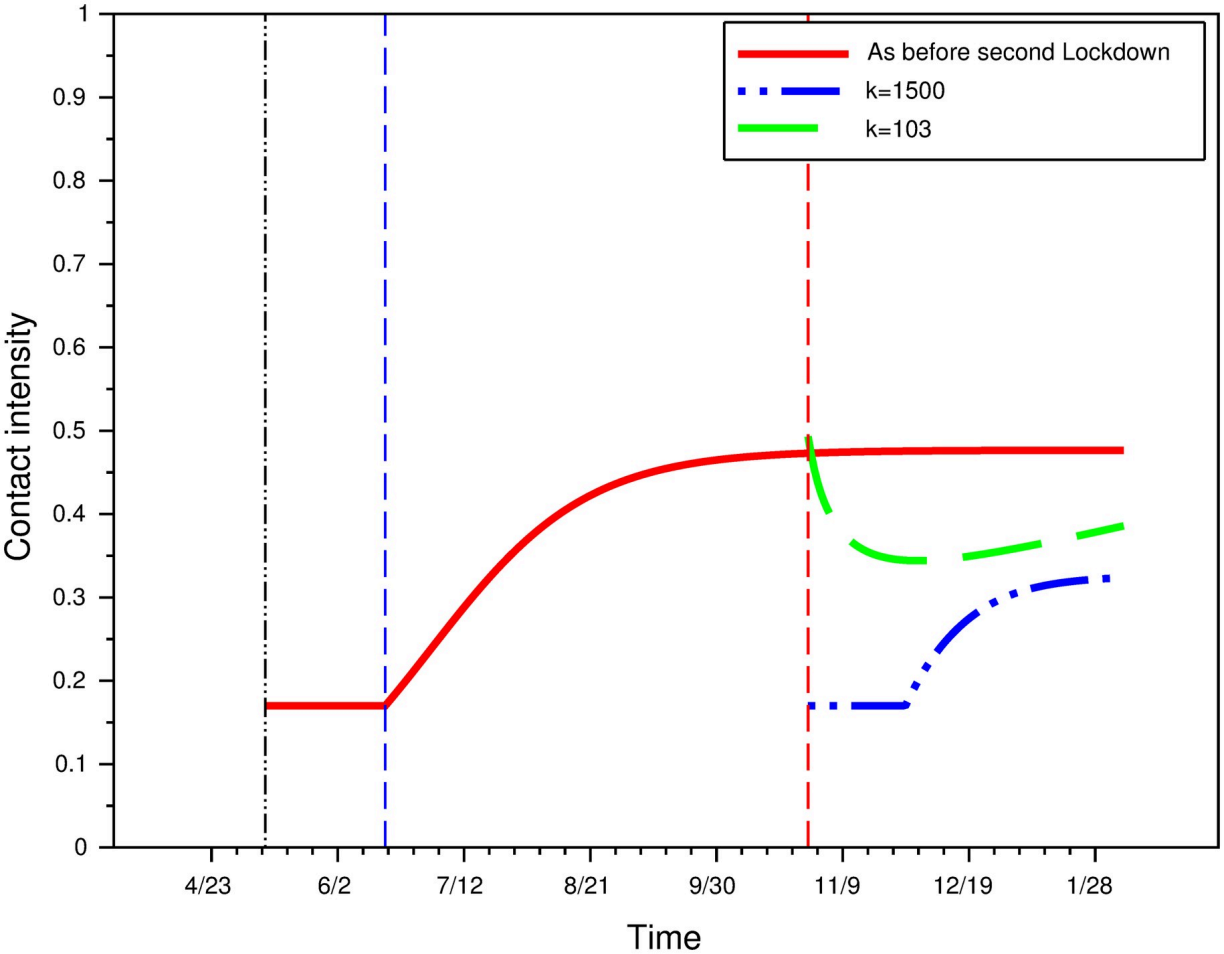
Contact intensity under various perception weights.

We consider that the rational contact intensity function as given by *φ*(*t*) = φ*(*L*;*k*) for *t* > *t*_4_, with *k* = 103 (the computed value at *t*_4_, beginning of second lockdown) and with *k* = 1500 (the computed value at *t*_2_, end of fist lockdown). Fig 10 represents contact intensity for these two values of *k* as well as the continuation of the function that applied before the second lockdown (*t*_4_, red vertical line). When *k* = 1500, contact intensity immediately drops to its minimal level (*a* = 0.1698953), meaning individuals exert as much effort as possible. It begins to increase once the reported incidence has lowered.

**Fig 11:**
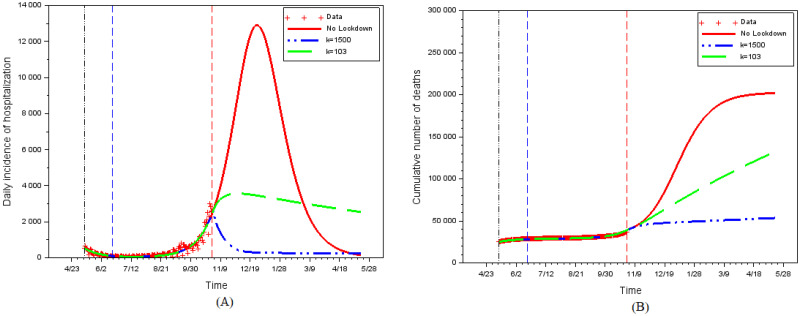
The impact of perceptions.

Fig 11 shows the impact of perception weights on the daily hospitalizations incidence and on the total number of deaths. In the absence of a second lockdown and with the continuation of the contacts intensity fitted on the data up to date *t*_4_, the peak of the epidemics would have been at the end of December 2020. As this second wave outbreak would have lasted several months, it would have led to a huge number of deaths (200,000 cumulative deaths from the start of the epidemics). In the rational behavioral model, individual equilibrium efforts enable to flatten the curve, but a perception weight of *k* = 103 (as was prevalent just before the second lockdown) is not enough to avoid a total number of deaths above 150,000. A much higher perception weight, such as *k* = 1500, is needed to avoid an epidemic peak after date *t*_4_.

#### Public salience

Survey data in [42] report that the main drivers of Japanese’s precautionary efforts have been the highly media-covered infection aboard the Diamond Princess cruise ship (February 2020), and governmental information, both corresponding to an increase in *k*. A potentially effective public policy consists in exacerbating public awareness about the disease, with media intervention and legal measures that draw attention to the infection risk. Variations in *k* can be used to represent the impact of media coverage and public discourse, as well as the expressive power of legal measures. This is a double-sword tool: Any relaxing of a legal constraint may be interpreted as a signal of a low infection risk, leading to a drop in *k*.

Importantly, effort can increase when the perception of infection risk increases (as measured by *k*) despite the lower epidemics spread associated with this higher effort. This is a specific advantage of the communication policy.

### Public policies partly crowd out individual efforts

We use our model to simulate the impact of various public policies on the COVID-19 epidemics, given individuals’ reaction to reported cases. The effectiveness of policies indeed strongly depends on the side impact they have on equilibrium self-protection efforts.

#### Partial lockdown

Imposing partial lockdown (for instance for vulnerable population or employees who have the ability to work remotely, or by closing some businesses such as bars and restaurants) helps reduce the cumulative number of deaths. We show however that its impact is lower than could be expected, once equilibrium rational choices are taken into account.

**Fig 12:**
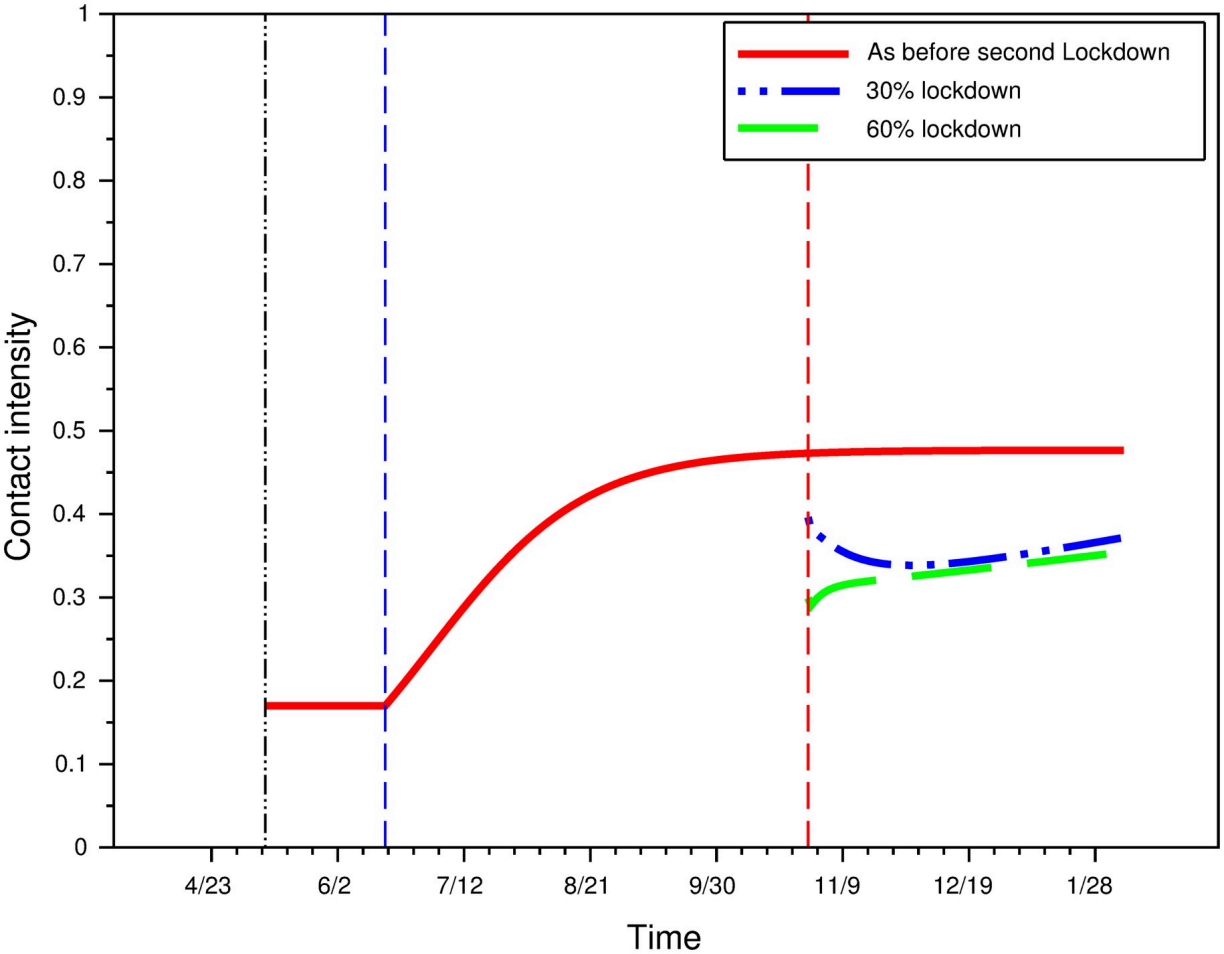
Impact of 30% and 60% partial lockdown on contact intensity.

Partial lockdown reduces infection prevalence, but this leads to increased contacts intensity for the proportion of individuals and/or activities not subjected to lockdown. Because there are fewer new cases than in the absence of any lockdown, effort decreases in the percentage of the population that remains under lockdown.

In our simulations, the direct distancing effect of lockdown still dominates, and both 30%- and 60%- partial lockdown reduce contacts intensity compared with no lockdown (Fig 12). However average contacts intensity is much more similar for the 3 scenarios considered than one would expect if one assumed behavior to be independent from prevalence. In particular the 30%- and 60%- scenarios yield nearly identical contact intensities after a few weeks. A sizable part of the potential benefits of partial lockdown thus disappears due to behavioral adjustments.

**Fig 13:**
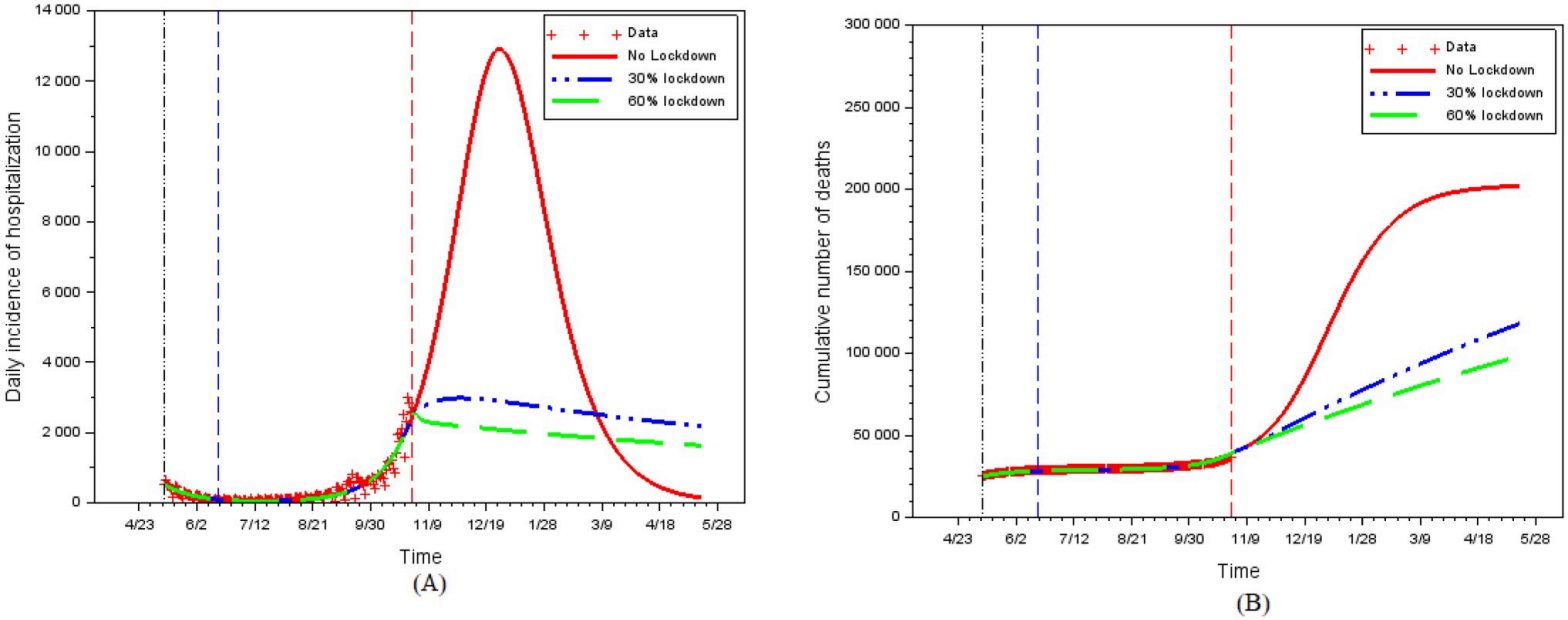
The impact of 30% and 60% partial lockdown. (A) Daily hospitalizations. (B) Cumulative number of deaths.

Partial lockdown is also not very effective at markedly reducing the number of deaths in the long run. A lockdown of 30% [resp. 60%] of the population or activities reduces the number of deaths to about 119,000 [resp. 100,000] instead of 200,000 (Fig 13). Despite locking in twice as many individuals (with the associated costs for the population), a 60% lockdown reduces the number of cumulative deaths by only 1/6th compared to a 30% lockdown.

For both a 30% and a 60% lockdown, the number of cumulative deaths is much lower than in the absence of restrictions, but remains very high. The benefit of partial lockdown comes from the fact that it (legally) imposes a low average contact intensity despite the decrease in prevalence. In our simulations, this strongly reduces hospitalizations and casualties. However, the crowding-out effect (reduced distancing effort) that arises on the activities or individuals not subjected to this partial lockdown reduces its effectiveness.

## Conclusion

Our results provide insight on public debates: Ignoring distancing choices likely leads to a strong overestimation (by a factor of 10) of the number of deaths avoided thanks to lockdown; but lockdown saves nearly twice as many lives as freely chosen efforts. Policies post-lockdown crowd out self-protection efforts so that their overall effectiveness is limited. Partial lockdown or business closure has much less impact than expected once behavioral adjustments are taken into account. Communication on the disease is a low-cost intervention that can increase distancing effort for given reported prevalence levels.

## Supporting information

S1 File List of epidemiological parameters. (PDF)

S2 File Refining the epidemiological model. (PDF)

S3 File Distancing effort by age group. (PDF)

S1 Fig (TIF)
