# Supplementary material for: The relative power of individual distancing efforts and public policies to curb the COVID-19 epidemics
Source: PLoS One. 2021 May 7;16(5):e0250764. doi: 10.1371/journal.pone.0250764 (PMC8104446; doi:10.1371/journal.pone.0250764)
Supplement: S1 File — (PDF) [file pone.0250764.s001.pdf]

The relative power of individual distancing efforts and public policies to curb the COVID-19 epidemics

S1 File. List of parameters

Symbol	Interpretation	Value
t_0	Time when the epidemic started	February 3rd [16]
t_1	Time when the lockdown started	March 17th
t_2	Time when the lockdown ends	May 11th
t_3	Time when intermediate lockdown ends	June 17th
t_4	Time when second lockdown started	October 30th
S_0	Number of susceptible at time t_0	$66.99.10^6$
L_0	Number of latent infectious at time t_0	3.675 [16]
A_0	Number of asymptomatic or mildly infectious cases at time t_0	0.892 [16]
I_0	Number of severe symptomatic infectious cases at time t_0	1
τ	Transmission rate at early stage	$4.23.10^{-9}$ [16]
γ	Death due to the disease	fitted
ν^{-1}	Average duration (days) of the asymptomatic period	7 [16]
f	Fraction of asymptomatic infectious who become reported symptomatic infectious	0.29
$\nu_1 = f\nu$	Rate at which asymptomatic infectious become reported symptomatic infectious	
$\nu_2 = (1-f)\nu$	Rate at which asymptomatic infectious become unreported symptomatic infectious	
η^{-1}	Average time symptomatic infectious have symptoms	7

Table 1. Table of epidemiological parameters. Numbers in brackets refer to the reference list (main article). Other parameters described at length in main article are not reported.
