# Supplementary material for: The relative power of individual distancing efforts and public policies to curb the COVID-19 epidemics
Source: PLoS One. 2021 May 7;16(5):e0250764. doi: 10.1371/journal.pone.0250764 (PMC8104446; doi:10.1371/journal.pone.0250764)
Supplement: S2 File — (PDF) [file pone.0250764.s002.pdf]

The relative power of individual distancing efforts and public policies to curb the COVID-19 epidemics

S2 File. Refining the epidemiological model

The exponential model, even though it fits our data quite well, does not entail a realistic representation of the time spent in each compartment [3]. It has been shown that, depending on the control measures, the exponential model can underestimate or overestimate the peak of an epidemics compared to other models with realistic compartments sojourn time distributions [1]. We study here whether individuals' perception of the epidemics is modified, when non-exponential compartments sojourn time distributions are considered.

Seminal papers in the 1990's have used differential integral equations [2]. When an Erlang distribution is considered, the chain trick enables to write the dynamics of the epidemics as an ODE.

Let us denote by T_L the random value representing the time spent in the latent compartment. Then assuming a 2-parameter Erlang distribution implies that $P\left(T_L \geq t\right) = \left(e^{-2\nu t}\left(1+2\nu t\right)\right)$. As in the exponential model, $\mathbb{E}\left[T_L\right] = \nu^{-1}$.

We also assume 2-parameter Erlang distributions for asymptomatic and hospitalized individuals. The epidemiological dynamical system is now given by

$$\begin{aligned}
S' &= -\tau_0 \varphi\left(t\right) S\left(L_1(t)+L_2(t)+A_1(t)+A_2(t)\right) \qquad (1)\\
L_1' &= \tau_0 \varphi\left(t\right) S\left(L_1(t)+L_2(t)+A_1(t)+A_2(t)\right) - 2\nu L_1\\
L_2' &= 2\nu L_1 - 2\nu L_2\\
I_1' &= 2f\nu L_2 - 2\left(\eta+\gamma\right) I_1\\
I_2' &= 2\left(\eta+\gamma\right) I_1 - 2\left(\eta+\gamma\right) I_2\\
A_1' &= \left(1-f\right) 2\nu L_2 - 2\eta A_1\\
A_2' &= 2\eta A_1 - 2\eta A_2
\end{aligned}$$

Perceived prevalence is now defined as $\widehat{P}_{er}\left(t\right) = k_{er}\left(t\right) 2f\nu L_2$. The perception weight $k_{er}\left(t\right)$ is computed by comparing the theoretical contact intensity φ^* with the contact intensity resulting from the 2-parameter Erlang model.

The perception weight $k\left(t\right)$ corresponding to the exponential model is compared to

$k_{er}(t)$ by computing

$$x(t) = \frac{k_{er}(t) - k(t)}{k(t)}$$

As shown in Fig. SF1, the perception weight is greater in the Erlang model than in the exponential model. The difference however sharply declines with prevalence. Fig. SF1 plots this difference over the period August-October 2020. In the summer, hospitalizations were quite rare in France, and their number increased until the second lockdown began at the end of October. The difference in the estimated perception weights steadily goes down over the period.

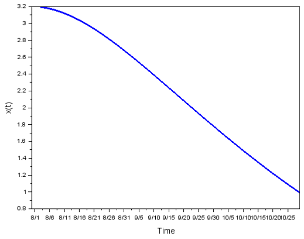

Fig. SF1. Spread $x(t)$ between perceptions weights computed with the Erlang model and the one computed in the exponential model.

References

1. Feng, Z. (2007). Final and peak epidemic sizes for SEIR models with quarantine and isolation. Mathematical Biosciences and Engineering, 4(4), 675.

2. van den Driessche, P. (1996). Some epidemiological models with delays. In Differential Equations and Applications to Biology and to Industry, 507-520. World Sci. Publishing, River Edge, NJ.

3. Wearing, H., Rohani, P., Keeling, M. (2005). Appropriate models for the management of infectious diseases. PLoS medicine, 2(7).
