# Supplementary material for: The relative power of individual distancing efforts and public policies to curb the COVID-19 epidemics
Source: PLoS One. 2021 May 7;16(5):e0250764. doi: 10.1371/journal.pone.0250764 (PMC8104446; doi:10.1371/journal.pone.0250764)
Supplement: S3 File — (PDF) [file pone.0250764.s003.pdf]

The relative power of individual distancing efforts and public policies to curb the COVID-19 epidemics

S3 File. Distancing effort by age group

Individuals in different age classes may have different opportunities, perceptions and preferences, and face different risks. Our analysis can be written for any number of age classes. However this increases greatly the number of parameters; the model becomes difficult to solve and the economic data to calibrate are not available. The theoretical model however provides a number of insights.

Three age classes

For simplicity, we consider that the population is decomposed into three classes: old adults, young adults and children. Effort is a choice for two classes, “young” (y) and “old” (o) adults, who are in proportions ρ^y and ρ^o. Children, in proportion $\rho^c = 1 - \rho^y - \rho^o$, are not able to choose their distancing behavior to a significant extent (e.g., going to school) and their distancing effort is fixed at some level ε^c. Mortality risks and general mobility differ significantly only after age 65 (for France and other richer countries), the age class we assimilate to o. Each age group has specific values for perception, risk aversion, effort costs, and disease severity. Equilibrium self-protection effort ε^a, $a = y, o$, is therefore now age specific. The average physical distancing effort in the population, $\overline{\varepsilon}$, is the average of the average effort in each age group: $\overline{\varepsilon} = \rho^y\varepsilon^y + \rho^o\varepsilon^o + (1 - \rho^y - \rho^o)\varepsilon^c$. Each individual takes it as given when making her decision, and considers her own impact on the average effort in her age group as null.

Older individuals are at higher risk if they get infected, so that $\lambda^o \leq \lambda^y$. They may also grant more attention to the disease risk and experience heightened fear, which would translate into a higher perceived prevalence parameter: $k^o \geq k^y$. Older individuals tend to be more risk averse, so that $\sigma^o \geq \sigma^y$ is a reasonable assumption.[1]

[1] Risk aversion however depends on many other factors [1], in particular gender and cognitive ability [2], and estimates by age classes are not available.

Individual distancing efforts and Nash equilibrium with different age classes

Each individual in age class a independently chooses her effort ε^a, taking the average effort $\overline{\varepsilon}$ as given, to maximize her expected utility:

$$\max_{\varepsilon^a}\{(1-(1-\varepsilon^a)\pi(\overline{\varepsilon},k^a,L)(1-\lambda^a))u(\varepsilon^a)\}$$

The best response from a single individual in class a, $a=y,o$, to average effort in the population is $BR^a(\overline{\varepsilon})=\min\{\max\{\varepsilon^{a*},0\},1-\varphi_{min}\}$, where ε^{a*} is the solution to the first-order condition of the maximization problem (and is the best response when it is interior to $[0,1-\varphi_{min}]$):

$$\varepsilon^{a*}\equiv\frac{1}{2\sigma^a-1}\left(\frac{\sigma^a}{\theta^a}-\frac{\sigma^a-1}{1-\lambda^a}\left[\frac{1}{k^a\tau_0(1-\overline{\varepsilon})fL}+\lambda^a\right]\right)$$

If the elderly face very low costs from distancing (θ^o close to 0), ε^{o*} will be above 1, and the best response is the maximum effort, 1.

In a symmetric Nash equilibrium, the average effort in the population is the average of efforts in each age class, knowing that all individuals in the same age class a choose the same best response effort $BR^a(\overline{\varepsilon})$: $\overline{\varepsilon}^*=\rho^c\varepsilon^c+\rho^yBR^y(\overline{\varepsilon}^*)+\rho^oBR^o(\overline{\varepsilon}^*)$. Assume that both $BR^y(\overline{\varepsilon}^*)$ and $BR^o(\overline{\varepsilon}^*)$ are interior (equal to ε^{y*} and ε^{o*}). The average equilibrium effort is the solution to

$$\begin{aligned}\overline{\varepsilon}^* &= \rho^c\varepsilon^c+\frac{\rho^y}{2\sigma^y-1}\left(\frac{\sigma^y}{\theta^y}-\frac{\sigma^y-1}{1-\lambda^y}\left[\frac{1}{k^y\tau_0(1-\overline{\varepsilon}^*)fL}+\lambda^y\right]\right)\\ &\quad+\frac{\rho^o}{2\sigma^o-1}\left(\frac{\sigma^o}{\theta^o}-\frac{\sigma^o-1}{1-\lambda^o}\left[\frac{1}{k^o\tau_0(1-\overline{\varepsilon}^*)fL}+\lambda^o\right]\right)\end{aligned}$$

provided that this solution lies in $[0,1-\varphi_{min}]$.

An interior solution is therefore solution to a second-degree equation of the form $A(\overline{\varepsilon}^*)^2+B\overline{\varepsilon}^*+C=0$, where

$$\begin{aligned}A &= 1\\ B &= -\Big(1+D-\frac{\rho^y(\sigma^y-1)}{2\sigma^y-1}\frac{\lambda^y}{1-\lambda^y}-\frac{\rho^o(\sigma^o-1)}{2\sigma^o-1}\frac{\lambda^o}{1-\lambda^o}\Big)\\ C &= D-\frac{\rho^y(\sigma^y-1)}{2\sigma^y-1}\frac{1}{(1-\lambda^y)k^y\tau_0fL}-\frac{\rho^o(\sigma^o-1)}{2\sigma^o-1}\frac{1}{(1-\lambda^o)k^o\tau_0fL}\\ D &= \rho^c\varepsilon^c+\frac{\rho^y}{\theta^y}\frac{\sigma^y}{2\sigma^y-1}+\frac{\rho^o}{\theta^o}\frac{\sigma^o}{2\sigma^o-1} \qquad (1)\end{aligned}$$

Because of the number of parameters, and of the possibility of multiple combinations of interior and non-inte, the number of cases to be considered is very large. We focus

below on the most relevant ones from an empirical point of view.

Severe or widespread disease for both young and old

We consider first the most relevant case in a situation of tension within ICUs, for instance, where the disease is worse for older individuals but is (perceived to be) severe for the young too, so that the perception weight k^a is very large and λ^a is very small, $a = y, o$. The same results hold when L is very large (widespread epidemics) and λ^a is very small (severe consequences).

Then $C = D$, $B = -(1 + D)$, and the discriminant of the second-degree equation simplifies to $1 - 2D + D^2 = (1 - D)^2$. The roots are D and 1.

Case 1: $D \geq 1$.

If $D \geq 1$, the equilibrium effort is characterized by

$$\begin{aligned} \overline{\varepsilon}^* &= 1 \\ \rho^y \varepsilon^{y*} + \rho^o \varepsilon^{o*} &= 1 - \rho^c \varepsilon^c \end{aligned}$$

From the expression of D, this situation is more likely if children are numerous and/or exercise much distancing ($\rho^c \varepsilon^c$ large). The equilibrium efforts from the young and the old are *substitutes*, and *decrease* in the weighted average effort of children, $\rho^c \varepsilon^c$. School closure, by increasing this weighted average, would lead to lower efforts from other age groups. Of course this result does not account for a major effect of school closure, which is that parents from young children may be forced to stay home.

Case 2: $1 - \varphi_{min} > D$.

Similarly, but with lower equilibrium effort levels than in case 1, if $1 - \varphi_{min} > D$, equilibrium effort is

$$\begin{aligned} \overline{\varepsilon}^* &= 1 - \varphi_{min} \\ \rho^y \varepsilon^{y*} + \rho^o \varepsilon^{o*} &= 1 - \varphi_{min} - \rho^c \varepsilon^c \end{aligned}$$

This case is more likely to happen if children are in lower proportion and/or exert little distancing.

As in case 1, ε^{y*} and ε^{o*} are substitutes and decrease in $\rho^c \varepsilon^c$.

Case 3: $1 - \varphi_{min} \leq D < 1$.

If $1-\varphi_{min} \leq D < 1$, equilibrium effort is characterized by

$$\begin{aligned} \bar{\varepsilon}^* &= D = \rho^c \varepsilon^c + \frac{\rho^y}{\theta^y}\frac{\sigma^y}{2\sigma^y-1} + \frac{\rho^o}{\theta^o}\frac{\sigma^o}{2\sigma^o-1} \\ \rho^y \varepsilon^{y*} + \rho^o \varepsilon^{o*} &= \frac{\rho^y}{\theta^y}\frac{\sigma^y}{2\sigma^y-1} + \frac{\rho^o}{\theta^o}\frac{\sigma^o}{2\sigma^o-1}. \end{aligned}$$

A solution is

$$\varepsilon^{a*} = \frac{\sigma^y}{\theta^y(2\sigma^y-1)} \quad \text{for } a = y, o$$

For an interior solution in the case of a severe disease, school closure has *no direct effect* on effort choices. The latter depend only on risk aversion and cost of distancing.

As could be expected, older individuals tend to choose higher effort levels than younger individuals, since their degree of risk aversion is higher ($\sigma^o \geq \sigma^y$).

Because average effort depends on proportions in each age group, older individuals, who exert more effort than younger ones, can afford to exert less distancing effort when they represent a higher percentage of the population. Elderly people may go out more in areas in which they are numerous, as they are aware that they will be surrounded by more prudent individuals on average.

Impact of children's effort.

To summarize, for a severe disease, a *public policy of school closure* would either *reduce* (cases 1 and 2) or have no impact (case 3) on individual effort. It can only have a positive impact by reducing transmission between children, and by forcing parents to remain home, as under partial lockdown.

Severe disease for the old only

Assume now that the disease is benign for younger individuals (λ^y close to 1) and that either k^y or L are low. The disease is however associated to a high perception and severe consequences for older individuals. Then the best response among the young adults is the minimal effort $1-\varphi_{min}$. Young adults do not respond to other individuals' effort in this context.

The equilibrium effort from the old is characterized by $\bar{\varepsilon}^* = \rho^c\varepsilon^c + \rho^y(1-\varphi_{min}) + \rho^o BR^o(\bar{\varepsilon}^*)$ for an interior average effort. Assuming that $BR^o(\bar{\varepsilon}^*) = \varepsilon^{o*}$ (interior solution), an interior average equilibrium effort is the solution to

$$\bar{\varepsilon}^* = \rho^c\varepsilon^c + \rho^y(1-\varphi_{min}) + \frac{\rho^o}{2\sigma^o-1}\left(\frac{\sigma^o}{\theta^o} - \frac{\sigma^o-1}{1-\lambda^o}\left[\frac{1}{k^o\tau_0(1-\bar{\varepsilon}^*)fL} + \lambda^o\right]\right)$$

We can show that the solution to the second-degree equation is increasing in $\rho^c\varepsilon^c$: in this context average effort responds to children's effort, although not by a ratio of 1.

Because younger individuals prefer to exert the minimal effort level, this set-up is structurally similar to one in which there is only one class who chooses effort. A difference with our main model with a single class is that the perceived risk (and therefore equilibrium choice) of older individuals depends on parameters that are exogenous from their point of view (that it: $\rho^c \varepsilon^c$ and $\rho^y(1 - \varphi_{min})$).

References

1. L'Haridon, O, Vieider, F (2019). All over the map: A worldwide comparison of risk preferences. Quantitative Economics, 10: 185-215.

2. Dohmen, T, Falk, A, Huffman, D, Sunde, U, Schupp, J, Wagner, G (2011). Individual risk attitudes: Measurement, determinants, and behavioral consequences. Journal of the European Economic Association, 9 (3): 522-550.
