# Supplementary figures and images for: The relative power of individual distancing efforts and public policies to curb the COVID-19 epidemics
Source: PLoS One. 2021 May 7;16(5):e0250764. doi: 10.1371/journal.pone.0250764 (PMC8104446; doi:10.1371/journal.pone.0250764)

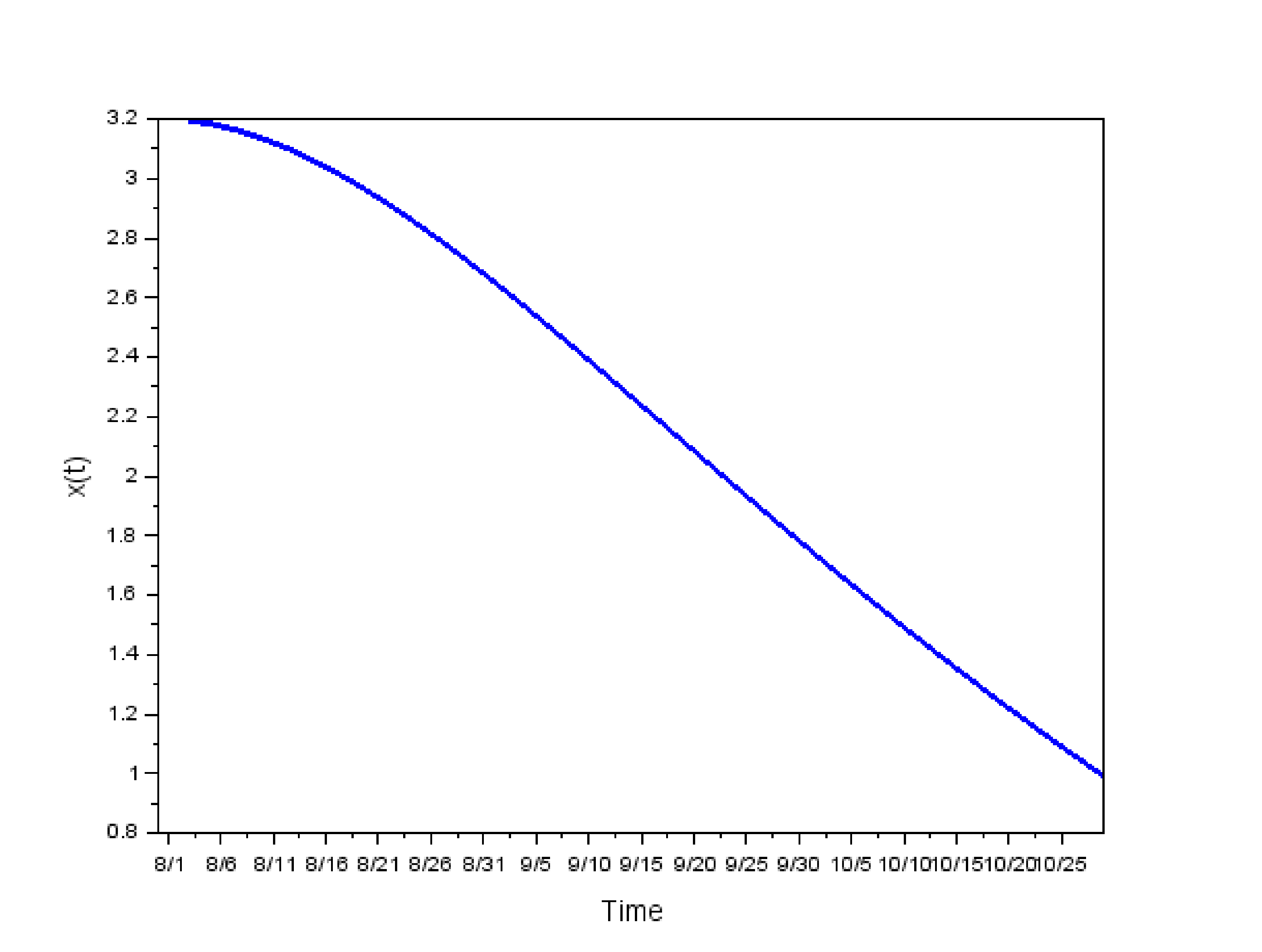

Supplement: S1 Fig — (TIF) [file pone.0250764.s004.tif]
